# Leveraging Synchrosqueezing Transform (SST)-based representations in a dual-stream attention framework to enhance sleep apnea detection and subtyping

**DOI:** 10.3389/fnins.2026.1853573

**Published:** 2026-05-29

**Authors:** Tianci Zhao, Cong Fu, Yang Li, Wei Chen, Huan Yu

**Affiliations:** 1 Department of Biomedical Engineering, College of Biomedical Engineering, Fudan University, Shanghai, China; 2 Department of Neurology, Huashan Hospital, Fudan University, Shanghai, China; 3 School of Biomedical Engineering, The University of Sydney, Sydney, NSW, Australia

**Keywords:** biomedical signal processing, computer aided diagnosis, deep learning, sleep apnea detection, time frequency analysis

## Abstract

**Introduction:**

Sleep apnea and hypopnea syndrome (SAHS) is a prevalent disorder with profound adverse effects on health and overall quality of life, thereby necessitating the development of accurate and accessible screening tools. Electrocardiogram (ECG)-based analysis, being non-invasive and readily deployable in low-cost hardware, offers a particularly convenient approach for SAHS screening and preliminary diagnosis. However, conventional time-frequency analysis often fails to capture the subtle yet critical patterns in ECG signals due to the Heisenberg uncertainty principle, leading to limited resolution and information loss.

**Methods:**

To overcome these limitations, this study proposes a Dual Stream Cross Attention Fusion Network (DSCAFNet) based on the uncertainty-mitigated time-frequency representations generated via Synchrosqueezing Transform (SST). The framework uniquely constructs two complementary, high-fidelity SST-based representations, which are strategically designed to provide distinct yet synergistic perspectives on the complex, non-stationary dynamics of SAHS. A dedicated cross-attention fusion module then harnesses these complementary views, enabling the model to discriminatively integrate multi-resolution features for significantly enhanced pattern recognition.

**Results:**

Extensively evaluated on the public Apnea-ECG dataset, DSCAFNet achieves an accuracy of 0.9572, a sensitivity of 0.9575, a specificity of 0.9584, and an F1-score of 0.9557, performing on par with state-of-the-art methods. More importantly, rigorous validation on a private Huashan-apnea dataset yields an accuracy of 0.9003 for binary classification and 0.7564 for four-class subtyping, demonstrating strong effectiveness and generalization.

**Conclusion:**

These consistent results across datasets highlight DSCAFNet as a promising framework for intelligent and accessible SAHS screening, with potential for integration into portable data acquisition systems combined with cloud-based analysis.

## 1 Introduction

Sleep apnea and hypopnea syndrome (SAHS) is a common sleep-disordered breathing characterized by recurrent episodes of cessation and resumption of breathing during sleep, affecting over a billion people worldwide (Benjafield et al., 2019). The clinical importance of accurately diagnosing and classifying SAHS arises from its diverse manifestations, with the syndrome being primarily categorized into obstructive sleep apnea (OSA), central sleep apnea (CSA), mixed sleep apnea (MSA) and hypopnea (Silvestri, 2018). Specifically, OSA originates from the physical collapse of the upper airway, CSA is caused by a dysfunction in the central nervous system’s respiratory drive, and MSA exhibits features of both. The pathophysiological differences among these types of sleep apnea lead to varied health risks, including oxygen desaturation and disrupted sleep architecture, which can adversely affect patients’ overall well-being. Therefore, accurate subtype classification is a prerequisite for tailoring subsequent diagnostic and therapeutic interventions (Bahrami and Forouzanfar, 2022). Currently, polysomnography (PSG) remains the clinical gold standard for sleep apnea (SA) diagnosis (Benjafield et al., 2019; Rundo and Downey III, 2019). However, the PSG monitoring process is complex, requiring multiple sensors (e.g., EEG, ECG, EOG) to be attached to the patient, which not only disrupts natural sleep but also increases costs and operational burden. Moreover, the reliance on experienced technicians for manual scoring of PSG data restricts its accessibility in community and home-based settings. Therefore, developing an automated, low-cost SA detection method based on fewer channels, or even a single channel, holds significant clinical and societal value.

Studies have shown that SA events trigger significant fluctuations in the autonomic nervous system and abnormalities in cardiac electrophysiology. These changes are reflected in the electrocardiogram (ECG) signal, specifically as alterations in the R-R interval (RRI), heart rate variability (HRV), and the morphology of the QRS complex (Khandoker et al., 2009). Consequently, single-channel ECG-based SA detection is considered a highly promising auxiliary diagnostic solution that can effectively simplify the diagnostic workflow (Fatimah et al., 2020). Early research primarily focused on extracting hand-crafted features from one-dimensional (1D) ECG signals. These methods typically utilized Heart Rate Variability (HRV) (Uddin et al., 2023; Varon et al., 2015) or frequency-domain analysis (Fatimah et al., 2020; Martín-Montero et al., 2021) to quantify the physiological changes caused by SA, which were then fed into traditional machine learning classifiers such as Support Vector Machines (SVM) (Ma et al., 2019), Linear Discriminant Analysis (LDA) (Song et al., 2015), and Random Forests (Rajesh et al., 2021; Sinha and Das, 2022). In recent years, with the advancement of deep learning, researchers have begun to employ end-to-end deep neural networks to learn discriminative features directly from 1D ECG signals. For instance, Shen et al. (2021) proposed a multi-scale deep network that used a dilated attention mechanism to weigh features from different receptive fields. Chen et al. (2023) fused RR Interval and RR Amplitude signal streams through a cross-attention module to enhance the network’s feature representation capabilities. However, 1D signals inherently struggle to fully capture the complex non-stationary and time-varying characteristics of ECG during SA events, which somewhat limits further improvements in model performance. To capture more comprehensive dynamic features of ECG signals, some studies have utilized time-frequency analysis (TFA) in conjunction with 2D Convolutional Neural Networks (2D-CNNs) for classification (Daubechies et al., 2011; Mashrur et al., 2021; Tang et al., 2025; Wang Y. et al., 2023). These approaches can simultaneously capture features in both the time and frequency domains. For example, Mashrur et al. (2021) used the Continuous Wavelet Transform (CWT) to generate time-frequency maps of ECG signals for SA detection. To further enhance the resolution of TFA, the Synchrosqueezing Transform (SST), an advanced post-processing technique, has been introduced into this field (Daubechies et al., 2011). SST compresses the blurred energy in the time-frequency spectrum onto the signal’s true instantaneous frequencies, thereby generating sharper and more concentrated time-frequency maps. Studies by Wang Y. et al. (2023) and Tang et al. (2025) have confirmed that SST-enhanced time-frequency maps can improve the robustness and effectiveness of SA detection.

**Figure 1:**
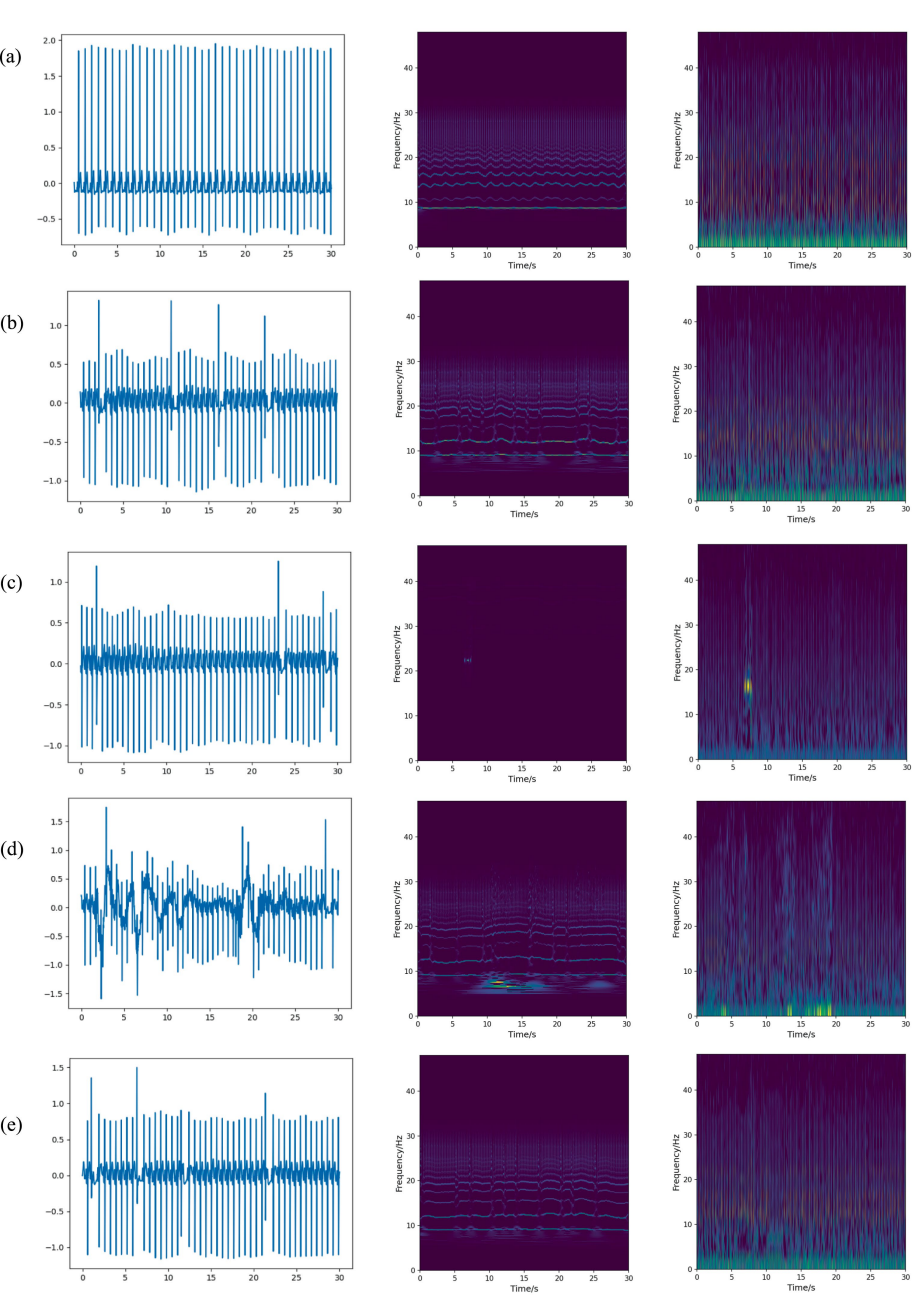
Illustration of five respiratory-related ECG signal categories and their corresponding time–frequency representations. Each row corresponds to a different class: **(a)** Normal, **(b)** OSA, **(c)** CSA, **(d)** MSA, and **(e)** HYP. Each column shows: raw ECG waveform, SST–CWT time–frequency representation, and SST–STFT time–frequency representation. This 5 × 3 panel highlights both morphological and spectral differences across sleep-related breathing patterns.

Despite the significant progress achieved by methods based on time-frequency analysis, existing studies still suffer from two primary limitations. First, the majority of current research focuses on the binary detection of sleep apnea (i.e., distinguishing apnea from normal breathing), while the multi-class classification of specific apnea subtypes (e.g., OSA, CSA, MSA) remains under-explored. Differentiating these subtypes is inherently more challenging due to the high inter-class similarity and the subtle nature of their physiological differences. Second, most existing methods rely solely on a single type of time-frequency transform (e.g., only CWT or STFT), which is often insufficient to fully capture the diverse characteristics of different apnea events. As illustrated in Figure 1, the raw ECG signals and their corresponding Synchrosqueezing Short-Time Fourier Transform (SST-STFT) and Synchrosqueezing Continuous Wavelet Transform (SST-CWT) spectrograms reveal that distinct apnea types exhibit unique spectral and transient patterns. A single representation may fail to extract the deep, discriminative features necessary to characterize the specific category of the apnea. The manifestation of SA events varies significantly across these transforms, highlighting the difficulty of exploiting complementary information. Therefore, effectively fusing multiple high-resolution time-frequency representations to maximize the extraction of discriminative features emerges as a critical research question.

Motivated by the analysis above, this paper proposes a Dual-Stream Cross-Attention Fusion Network (DSCAFNet), a novel deep learning framework designed for robust sleep apnea detection and subtype classification. The core of DSCAFNet lies in its synergistic use of synchrosqueezed time-frequency representations (SST-enhanced CWT & STFT) and a dual-stream architecture with cross-attention fusion, enabling complementary and semantically feature learning. The main contributions are summarized as follows:

1) We demonstrate the efficacy of SST in sharpening the time-frequency representations of conventional CWT and STFT. By capturing the fine-grained oscillatory dynamics inherent to apnea events, these enhanced representations improve the discriminability among obstructive, central, and mixed sleep apnea subtypes, which are typically challenging to distinguish.
2) We propose a dual-stream deep learning framework that processes time frequency representations in parallel to capture complementary spectral-temporal characteristics intrinsic to different transforms. Additionally, a cross-attention fusion module is introduced to integrate heterogeneous time–frequency cues at a deeper semantic level, moving beyond simple concatenation or averaging.
3) We conduct a comprehensive evaluation on both the public Apnea-ECG dataset and private Huashan-Apnea dataset. The proposed model achieves excellent performance in both binary (SA/Normal) and multi-class (OSA/CSA/MSA/HYP) classification tasks, thereby demonstrating its effectiveness and generalization capability.

## 2 Materials and methods

**Figure 2:**
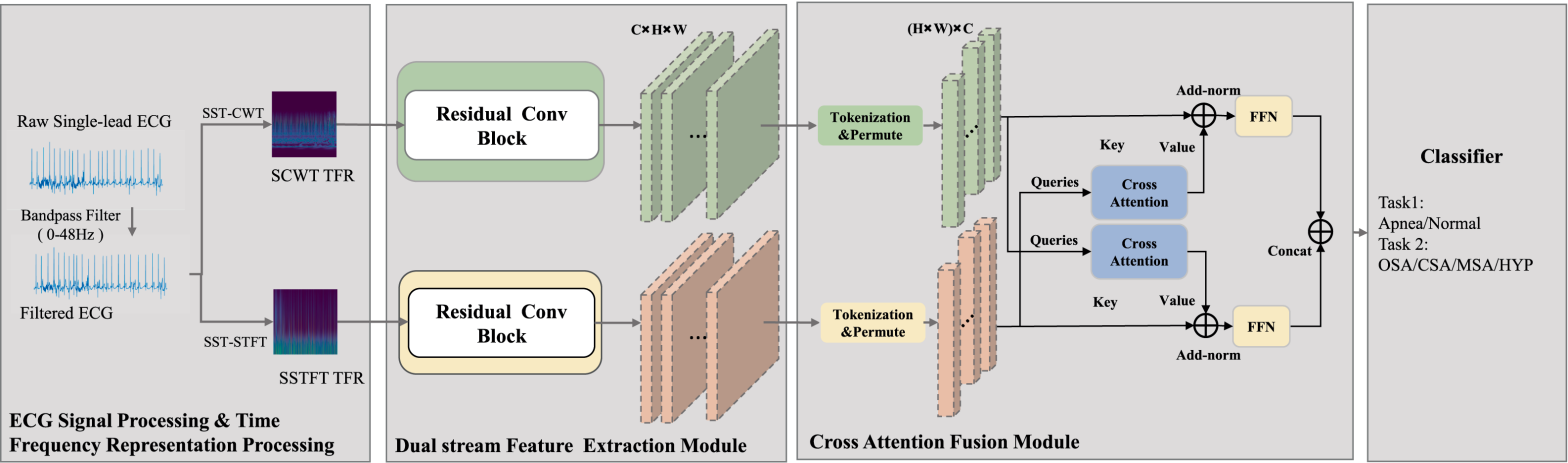
Overview of our proposed architecture using time frequency representations.

Figure 2 shows the overall pipeline of SA detection in this study mainly consisting of two steps: signal preprocessing and SA modeling, which are described in detail as follows.

### 2.1 Dataset

In this paper, two datasets were used to validate the method. A brief overview of both the datasets are as follows:

1) Apnea-ECG dataset: To evaluate the performance of the proposed model, this study utilized the public Apnea-ECG dataset (Penzel et al., 2000), available on PhysioNet. This dataset comprises 70 single-lead ECG recordings from 32 subjects (25 males and 7 females), collected by the Philipps University of Marburg, Germany. The specific information is listed in Table 1. The ECG signals were sampled at 100 Hz, with recording durations ranging from 6.7 to 9.6 h. Each one-minute segment of the ECG recordings was annotated by expert physicians, where the label “A” indicates an apneic event and “N” denotes a normal breathing period. It is noteworthy that the dataset does not differentiate between various types of respiratory events; all obstructive, central, and mixed apneas, as well as hypopneas, are uniformly labeled as “A”.
2) Huashan-Apnea dataset: To further validate the effectiveness of our proposed method, we collected a private sleep apnea dataset, hereinafter referred to as Huashan-Apnea. This dataset contains overnight polysomnography (PSG) recordings from 133 subjects (99 males and 9 females), gathered at Huashan Hospital, Fudan University. The data were annotated by expert physicians, with annotations detailing the occurrence and duration of various respiratory events, including Obstructive sleep apnea (OSA), Central sleep apnea (CSA), Mixed sleep apnea (MSA), and Hypopnea. The ECG signals were sampled at 512 Hz, and the recording durations range from 5.8 to 10.5 h. Informed consent was obtained from all participants, and the study was approved by the Institutional Review Board (IRB) of Huashan Hospital, Fudan University (Ethics approval number: 2021-811-X1). The Huashan-Apnea dataset contains multiple types of sleep apnea events, the specific epoch counts of which are detailed in Table 2.

**Table 1:** Details of apnea ECG dataset.

Dataset description	Apnea-ECG	Huashan-Apnea
Gender (Male/Female)	57/13	123/10
Age	46.82 ± 11.73 (27.0–63.0)	47.1 ± 14.9 (5–80)
BMI	28.88 ± 6.51 (19.2–45.3)	42.0 ± 16.4 (15.7–39.8)
AHI	26.41 ± 27.85 (0.00–93.50)	26.7 ± 16.0 (1.50–101.8)
Record Time (one night)	493.50 ± 31.48 (401.00–558.00)	508.82 ± 49.67 (350.5–630.3)

**Table 2:** The number of different type of sleep apnea events.

Dataset	Normal	Sleep apnea			
Apnea-ECG/ epochs	21,527	12,826			
Huashan-Apnea/ epochs	Normal	OSA	CSA	MSA	HYP
	111,748	7,135	3,256	4,576	6,047

### 2.2 ECG signal processing

ECG signals are susceptible to various types of noise and artifacts, such as electrode motion artifacts, muscle artifacts, random noise, and baseline wander, which necessitate signal filtering and segmentation (Sörnmo and Laguna, 2005; Zarei et al., 2022). Therefore, we first applied a bandpass filter with a passband of 0–48 Hz to remove these disturbances. This frequency range was chosen to maximally preserve the essential information within the ECG signal, including the P-wave, QRS complex, and T-wave, as these components are physiologically correlated with sleep apnea events.

Subsequently, the signals from the Apnea-ECG and Huashan-Apnea datasets were segmented into 60-s and 30-s epochs, respectively. Furthermore, as the datasets were recorded at different sampling rates, all ECG signals were downsampled to a uniform frequency of 100 Hz to ensure consistency.

Moreover, prior literature has demonstrated that incorporating adjacent segments can significantly enhance sleep apnea detection performance (Chen et al., 2021). Generally, the physiological characteristics of human apnea or normal breathing tend to persist for a certain duration during sleep, rather than switching rapidly between apneic and normal states. Consequently, information from adjacent segments can help mitigate the problem of insufficient information within a single epoch. To leverage this temporal context, the spectrogram for each epoch was computed by combining it with its preceding and succeeding epochs, thereby enriching the information content for the central segment under analysis.

Due to the different epoch lengths (60 s for Apnea-ECG and 30 s for Huashan-Apnea), the resulting time-frequency representations (TFRs) naturally have different temporal dimensions. To ensure a consistent input size for the neural network, all TFRs were resized to 224 × 224 × 3 using LANCZOS-based image resampling.

This process effectively normalizes the spatial resolution of the TFRs, acting as interpolation for shorter epochs and compression for longer epochs. It should be noted that the primary frequency characteristics are determined during the SST-based transformation stage, and the subsequent resizing mainly affects the image resolution rather than the intrinsic spectral information.

### 2.3 Time frequency representation processing

#### 2.3.1 Overview of Synchrosqueezing transform

A non-stationary signal s(t) can be modeled as the sum of multiple amplitude-modulated and frequency-modulated (AM-FM) components as Equation 1:

$$s(t)=\sum_{k=1}^{N}A_{k}(t)e^{i2\pi \phi_{k}(t)}$$ (1)

where $A_{k}(t)$ is the instantaneous amplitude of the k-th component, and $f_{k}(t)=\phi_{k}^{\prime }(t)$ is its instantaneous frequency (IF). The objective of SST is to precisely extract these frequency trajectories from a smeared Time Frequency Representation (TFR).

*Instantaneous frequency calculation and energy reassignment*: For each non-zero point T(t,η) in a TFR ω(t,η) , an instantaneous frequency estimate is computed. This estimate is obtained from the phase derivative of ω(t,η) with respect to time as Equation 2 (Daubechies et al., 2011):

ω(t,η)=Re{12πi∂∂tT(t,η)T(t,η)}forT(t,η)≠0 (2)

This calculation estimates the frequency to which the energy at the time-frequency point T(t,η) is closest.

*Energy squeezing*: The final synchrosqueezed representation S(t,ω) is generated. This process squeezes the energy from the original TFR T(t,η) at an analysis frequency η and reassigns it to a new, more precise instantaneous frequency ω . Its mathematical expression is defined in Equation 3:

S(t,ω)=∫T(t,η)·δ(ω−ω(t,η))dη (3)

where δ(·) is the Dirac delta function. In calculation, this is implemented as a summation process: the energy contributions $T(t,\eta_{k})$ are summed over all analysis frequencies whose corresponding instantaneous frequency $\omega (t,\eta_{k})$ falls into a specific frequency bin centered at $\left[\omega_{l}-\Delta \omega /2,\omega_{l}+\Delta \omega /2\right]$ . This sum is then assigned to the new TFR at point $\left(t,\omega_{l}\right)$ .

#### 2.3.2 Synchrosqueezing STFT TFRs

When SST is applied to the Short-Time Fourier Transform (STFT), the steps and calculation equations (Equations 4–6) are as follows (Oberlin et al., 2014):

a) *STFT Calculation*: For a signal s(t) , its STFT is defined as:

$$V_{g}(t,f)=\int_{-\infty }^{\infty }s(\tau )g(\tau -t)e^{-i2\mathrm{\pi f\tau}}\;\mathrm{d\tau}$$ (4)

where g(t) is a window function, t is time, and f is frequency. The result $V_{g}(t,f)$ is a complex-valued time-frequency matrix.

b) *Instantaneous Frequency Calculation*: Based on the STFT result Ss(t,f)Ss​(t,f), the instantaneous frequency is estimated as:

ωs(t,f):ωs(t,f)=Re{12πi∂∂tVg(t,f)Vg(t,f)} (5)

Here, $\omega_{s}(t,f)$ estimates the true instantaneous frequency corresponding to the energy at the point (t,f) .

c) Synchrosqueezing: The energy in the STFT spectrum is reassigned to obtain the SST result $S_{s}(t,\omega )$ :

$$S_{s}(t,\omega )=\int_{-\infty }^{\infty }V_{g}(t,f)\cdot \delta (\omega -\omega_{s}(t,f))\;\mathrm{df}$$ (6)

Through this process, the frequency smearing caused by the windowing effect in the STFT is significantly reduced, and the energy is concentrated onto the true frequency trajectories of the signal’s components.

#### 2.3.3 Synchrosqueezing CWT TFRs

a) CWT Calculation: For a signal s(t) , its Continuous Wavelet Transform (CWT) is defined as Equation 7:

$$W_{\psi }(t,a)=\frac{1}{\sqrt{a}}\int_{-\infty }^{\infty }s(\tau )\psi^{*}(\frac{\tau -t}{a})\;\mathrm{d\tau}$$ (7)

where ψ(t) is the mother wavelet, a>0 is the scale parameter, and t is time. The result $W_{\psi }(t,a)$ is a complex-valued time-scale matrix. In this work, the Morlet wavelet is used for the analysis.

b) Instantaneous Frequency Calculation: Based on the CWT result $W_{\psi }(t,a)$ , the instantaneous frequency is estimated as Equation 8:

ωw(t,a):ωw(t,a)=Re{12πi∂∂tWψ(t,a)Wψ(t,a)} (8)

This frequency $\omega_{w}(t,a)$ estimate maps the scale a to an instantaneous frequency, a mapping that depends on the center frequency of the mother wavelet.

c) Synchrosqueezing: The energy from the time-scale plane is reassigned to the time-frequency plane to obtain the SST result $S_{w}(t,\omega ):$
.

Its mathematical expression is defined in Equation 9:

$$S_{w}(t,\omega )=\int_{0}^{\infty }W_{\psi }(t,a)\cdot a^{-3/2}\cdot \delta (\omega -\omega_{w}(t,a))\;\mathrm{da}$$ (9)

CWT-based SST effectively sharpens the energy diffusion along the scale axis caused by the wavelet basis function, thereby generating a highly concentrated time-frequency representation.

All sleep apnea signal segments (derived from the ECG signals) were processed using both SST-STFT and SST-CWT to generate time-frequency maps. These maps were then output as 224 × 224 × 3 images, as illustrated in Figure 1.

### 2.4 Network architecture

To accurately identify different types of sleep apnea events, we propose a novel Dual Stream Cross Attention Fusion Network (DSCAFNet). As illustrated in Figure 2, DSCAFNet comprises three main modules: a Dual Stream Feature Extraction Module (DSFEM), a Cross-Attention Fusion Module (CAFM), and a final classifier. The network operates by first using the DSFEM to parallelly extract spatial feature maps from two time-frequency images. Subsequently, the CAFM mutually enhances the information between the two feature streams via a cross-attention mechanism, thereby significantly improving the network’s sensitivity to critical discriminative features of different SA types. The enhanced feature maps from the CAFM are then fed into the classifier, which outputs the predicted labels through a linear layer to perform the final classification.

Our model is based on the core principle that the characteristics of SA events often manifest as subtle differences in time-frequency maps. A single feature representation may be insufficient to capture these nuanced variations, especially in patients with mild symptoms. Our approach addresses this by independently extracting features from two distinct time-frequency representations via a dual stream architecture and then deeply fusing these representations through an interactive module designed to amplify the critical class-discriminative information.

**Figure 3:**
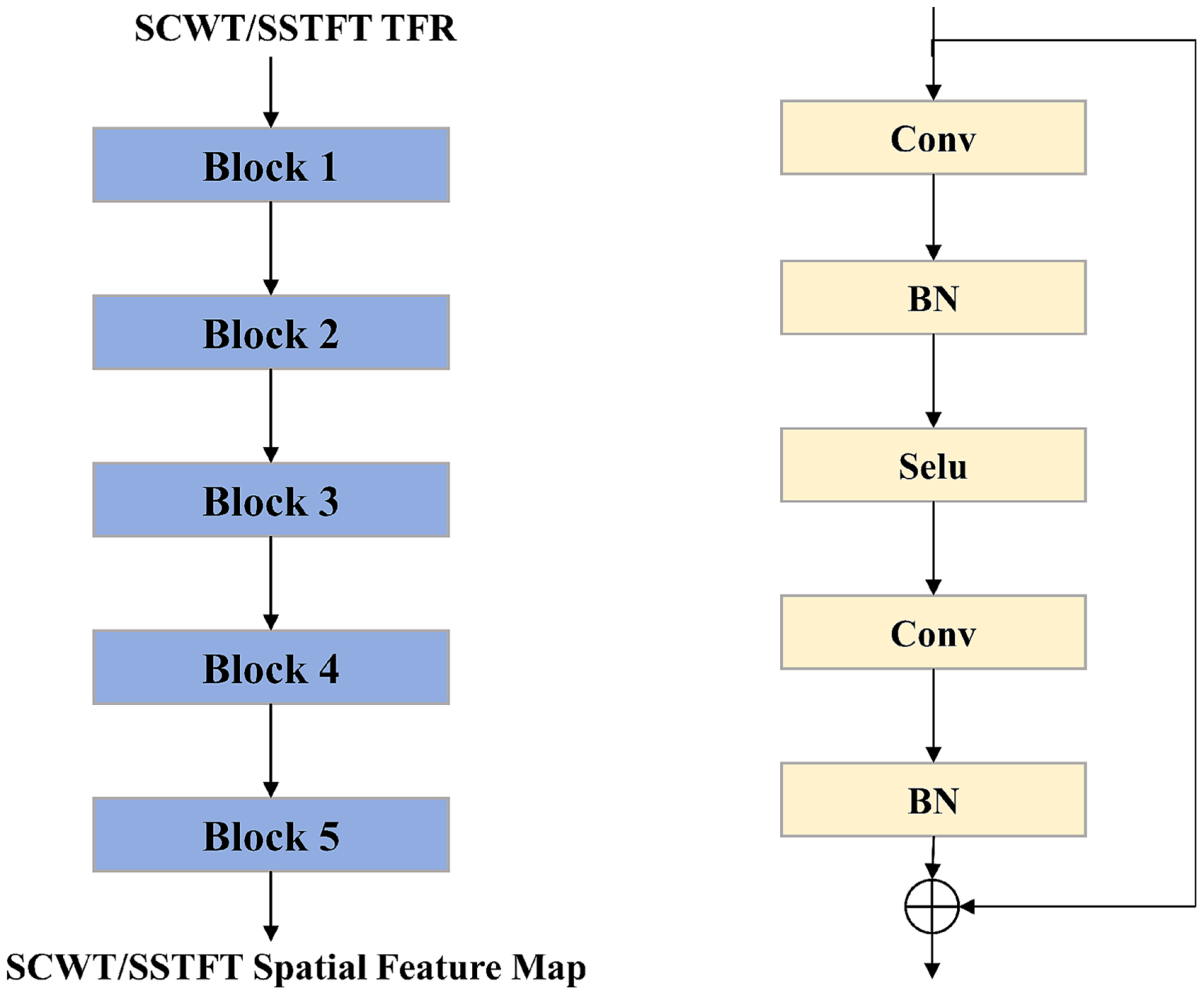
Detailed structure of DSFEM, left is SCWT/SSTFT stream feature extractor and right is the basic block of DFSEM.

a) Dual Stream Feature Extraction Module (DSFEM): For some SA events, particularly those with mild symptoms, the patterns in the time-frequency maps can be highly non-salient and easily obscured by background signals. To address this, we designed a dual stream feature extractor to process two different TF representations, $X_{\text{SCWT}}$ and $X_{\text{SSTFT}}$ , which SCWT and SSTFT refer to SST-CWT and SST-STFT respectively, derived from the same ECG signal. Let these inputs be $X_{\text{SCWT}},X_{\text{SSTFT}}\in R^{B\times C\times H\times W}$ , where C is the number of channels, and H and W are the height and width of the time-frequency maps. The detailed structure show in Figure 3 and detailed network parameters is in Table 3. The corresponding equations are described in Equations 10–14.

**Table 3:** The detailed configuration of the proposed DSCAFNet.

Module	Layer type	SCWT stream	SSTFT stream
DSFEM	Conv+BN + Selu+MaxPool	filter size = 7 × 7, filter = 64 stride = 2,padding = 3	filter size = 7 × 7, filter = 64 stride = 2, padding = 3
	2 × BasicBlock	filter size: 3 × 3,filter = 64, stride = 2, padding = 1	filter size: 3 × 3,filter = 64, stride = 2, padding = 1
	2 × BasicBlock	filter size: 3 × 3, filter = 128, stride = 2, padding = 1	filter size: 3 × 3, filter = 128, stride = 2, padding = 1
	2 × BasicBlock	filter size: 3 × 3, filter = 256, stride = 2, padding = 1	filter size: 3 × 3, filter = 256, stride = 2, padding = 1
	2 × BasicBlock	filter size: 5 × 5, filter = 512, stride = 2, padding = 2	filter size: 5 × 5, filter = 512, stride = 2, padding = 2
Cross Attention Fusion	Multihead Attention	dq = dk = dv = 512, Attention heads = 4	
Classifier	AvgPooling	—	
	Linear	Input size = 1,024, output size = 512	
	Selu	—	
	Linear	Input size = 512, output size = 2/4	

Each time-frequency map is fed into an independent, residual CNN-based backbone. The design, based on multi-layer residual convolutions, effectively builds a deep network capable of extracting hierarchical spatial feature maps, ranging from low-level textures to high-level semantics. This deep feature extraction capability is crucial for capturing subtle event patterns. The DSFEM outputs two spatial feature maps, $F_{\text{SCWT}}$ and $F_{\text{SSTFT}}$ , represented as follows:

$$F_{\text{SCWT}}=f^{\text{SCWT}}(X_{\text{SCWT}}),F_{\text{SSTFT}}=f^{\text{SSTFT}}(X_{\text{SSTFT}})$$ (10)

where $f^{\text{SCWT}}(\cdot )$ and $f^{\text{SSTFT}}(\cdot )$ represent the network backbones for the CWT and STFT streams, respectively. The output feature maps have dimensions $F_{\text{SCWT}},F_{\text{SSTFT}}\in R^{B\times C'\times H'\times W'}$ .

By employing a parallel dual stream approach, we not only process two different input representations but also introduce beneficial redundancy during the feature extraction phase. This means that even if the features in one stream are ambiguous, information from the other stream can provide complementary cues, thereby improving the overall robustness and accuracy of the detection.

b) Cross-Attention Fusion Module (CAFM): Traditional fusion methods often rely on simple concatenation or element-wise addition of feature maps, which are inadequate for modeling the complex, non-linear dependencies between them. To address the challenge of effective information interaction between the two time-frequency streams, we introduce a fusion module based on cross-attention. This module enables the two feature streams to perform fine-grained information exchange and mutual enhancement at the token level, significantly boosting the network’s sensitivity to key discriminative features.

We first convert the 2D feature maps, $F_{\text{SCWT}}$ and $F_{\text{SSTFT}}$ , output by the feature extractor into 1D token sequences. This is achieved by flattening the spatial dimensions, resulting in two token sequences, $T_{\text{SCWT}}$ and $T_{\text{SSTFT}}$ :

$$\begin{array}{rr} T_{\text{SCWT}} & =\text{Permute}(\text{Tokenization}(F_{\text{SCWT}}))\\ T_{\text{SSTFT}} & =\text{Permute}(\text{Tokenization}(F_{\text{SSTFT}})) \end{array}$$ (11)

where $T_{\text{SCWT}},T_{\text{SSTFT}}\in {\mathbb{R}}^{B\times L\times D}$ with L=H×W being the sequence length and $D=C^{\prime }$ being the embedding dimension of each token. Each token can be viewed as an abstract semantic representation of a local region in the original time-frequency map.

This is the key step for information interaction. We employ a cross-attention mechanism where one sequence serves as the Query (Q), while the other sequence provides the Key (K) and Value (V). This allows the query sequence to attend to all tokens in the key/value sequence and aggregate information based on relevance using a multi-head attention mechanism.

$$\text{Attn}(Q,K,V)=\text{softmax}(\frac{QK^{⊤}}{\sqrt{d_{k}}})V$$ (12)

This process is bidirectional: $T_{\text{SCWT}}$ queries $T_{\text{SSTFT}}$ to generate an enhanced sequence $T_{\text{SCWT}}^{\text{attn}}$ that is informed by $T_{\text{SSTFT}}$ . $T_{\text{SSTFT}}$ queries $T_{\text{SCWT}}$ to generate an enhanced sequence $\;T_{\text{SSTFT}}^{\text{attn}}$ that is informed by $T_{\text{SCWT}}$ :

$$\begin{array}{cc} T_{\text{SCWT}}^{\text{attn}} & =\text{Attn}(T_{\text{SCWT}},T_{\text{SSTFT}},T_{\text{SSTFT}}),\\ T_{\text{SSTFT}}^{\text{attn}} & =\text{Attn}(T_{\text{SSTFT}},T_{\text{SCWT}},T_{\text{SCWT}}) \end{array}$$ (13)

$$\begin{array}{cc} {\hat{T}}_{\text{SCWT}} & =\mathrm{FFN}(\text{LayerNorm}(T_{\text{SCWT}}+T_{\text{SCWT}}^{\text{attn}})),\\ {\hat{T}}_{\text{SSTFT}} & =\mathrm{FFN}(\text{LayerNorm}(T_{\text{SSTFT}}+T_{\text{SSTFT}}^{\text{attn}})) \end{array}$$ (14)

This bidirectional mechanism ensures that the information exchange is symmetric and comprehensive, allowing the two feature streams to mutually calibrate and complement each other. Through this interactive fusion module, we obtain two feature sequences, ${\hat{T}}_{\text{SCWT}}$ and ${\hat{T}}_{\text{SSTFT}}$ , that both retain their unique characteristics and are enriched with information from the complementary stream. This provides a high-quality feature representation for the final classification task.

### 2.5 Data augmentation

Since traditional geometric data augmentation techniques, such as rotation and flipping, disrupt the physical meaning of time-frequency representations, we employed SpecAugment (Park et al., 2019), a method specifically designed for spectrogram analysis, to augment the training data. This method improves the model’s robustness to the partial loss of information by applying masks to the input features. Our augmentation pipeline consists of the following two core operations:

*Time Masking*: A contiguous block of time steps (ranging from 0 to 20 pixels) is randomly selected and masked along the time axis (horizontal axis). This operation simulates a temporary interruption or noise artifact in the signal, forcing the model to learn from contextual information rather than relying on features at a single point in time.

*Frequency Masking*: A contiguous range of frequency channels (ranging from 0 to 20 pixels) is randomly selected and masked along the frequency axis (vertical axis). This operation simulates noise or signal attenuation within a specific frequency band, encouraging the model to utilize a broader range of spectral information for classification.

All augmentation techniques were applied exclusively to the training set. The validation and test sets remained unaugmented to ensure a fair and unbiased evaluation of the model’s performance.

## 3 Experiment settings

### 3.1 Implementation details

Our experiments were implemented using PyTorch with Python 3.8, and conducted on a workstation equipped with two RTX 3060 GPUs and 24 GB of RAM.

To ensure a robust and unbiased evaluation, a subject-independent five-fold cross-validation strategy was adopted. Specifically, all data from each subject were assigned exclusively to a single fold, preventing any overlap of subjects between the training and validation sets and thereby avoiding potential data leakage. The dataset was partitioned into five mutually exclusive subject groups. In each fold, four groups (approximately 80% of the subjects) were used for training, while the remaining group (approximately 20%) was used for validation. This process was repeated five times so that each group served as the validation set exactly once.

Model optimization was performed using stochastic gradient descent (SGD), with model weights re-initialized at the beginning of each fold. All experiments were conducted using consistent hyperparameter settings, including an initial learning rate of 0.001, a batch size of 32, and 20 training epochs.

Considering the class imbalance between sleep apnea (SA) and normal samples, a weighted cross-entropy loss function was employed.

### 3.2 Metrics

To comprehensively evaluate the classification performance of the proposed model, we employed several commonly used statistical metrics, including Accuracy (Acc), Specificity (Spec), Sensitivity (Sens), and F1-score. These metrics were computed based on the confusion matrix elements, namely True Positive (TP), True Negative (TN), False Positive (FP), and False Negative (FN).

In the binary classification experiments, the model was trained to distinguish between normal. The metrics are defined in Equations 15–19.

$$\text{Accuracy}=\frac{\mathrm{TP}+\mathrm{TN}}{\mathrm{TP}+\mathrm{TN}+\mathrm{FP}+\mathrm{FN}}$$ (15)

$$\text{Precision}=\frac{\mathrm{TP}}{\mathrm{TP}+\mathrm{FP}}$$ (16)

$$\begin{array}{c} \text{Sensitivity}=\frac{\mathrm{TP}}{\mathrm{TP}+\mathrm{FN}} \end{array}$$ (17)

$$\text{Specificity}=\frac{\mathrm{TN}}{\mathrm{TN}+\mathrm{FP}}$$ (18)

$$F1-\text{score}=\frac{2\times \mathrm{TP}}{2\times \mathrm{TP}+\mathrm{FP}+\mathrm{FN}}$$ (19)

and sleep apnea (SA) samples. Consequently, all the aforementioned performance metrics were computed for each class (Normal and SA) individually to evaluate the model’s discriminative capability between these two states. The final reported figures represent the average performance across all test samples.

In the multi-class experiments, the model was designed to classify four types of sleep-disordered breathing events: Obstructive sleep apnea (OSA), Central sleep apnea (CSA), Mixed sleep apnea (MSA), and Hypopnea (HYP). For this task, the metrics were calculated for each class separately using a one-vs-rest strategy.

The overall F1-score reported in this study corresponds to the Macro-F1 score, which is calculated by averaging the F1-scores of all classes and is more suitable for evaluating performance under class imbalance.

## 4 Results

### 4.1 Overall performance

Table 4 present the five-fold cross-validation results of our proposed method for the binary and four-class classification tasks, respectively.

**Table 4:** Five fold validation results of 2-class classification (Normal/Apnea) in both datasets.

Dataset	Task	Acc	Sens	Spec	F1-score
Apnea-ECG	2-classs Classification	0.9557 ± 0.0072	0.9572 ± 0.0064	0.9575 ± 0.0058	0.9584 ± 0.0059
Huashan-Apnea	2-class Classification	0.9003 ± 0.0049	0.8814 ± 0.0055	0.8314 ± 0.0075	0.8473 ± 0.0115
	4-class Subtyping	0.7564 ± 0.0117	0.7546 ± 0.0107	0.9117 ± 0.0061	0.7497 ± 0.0084

**Figure 4:**
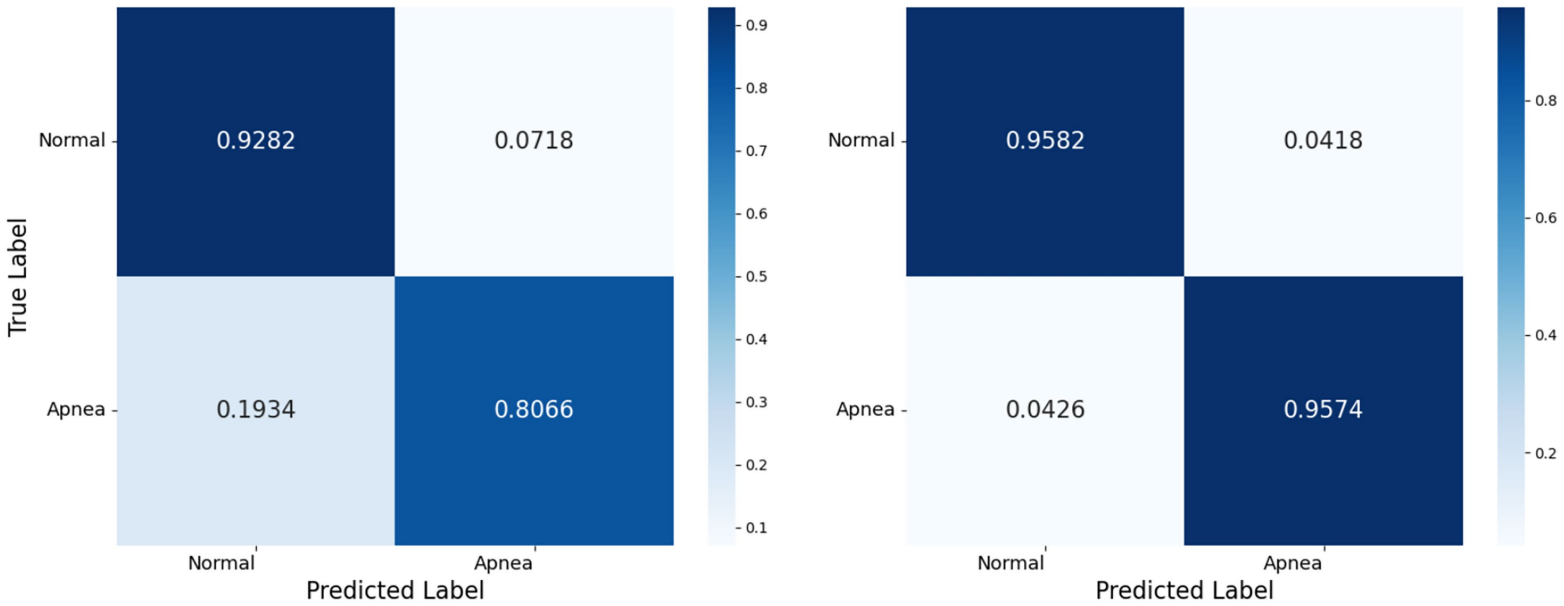
All fold confusion matrix in 2-class classification task.

Overall performance in sleep apnea detection: As shown in Table 4 and Figure 4, our DSCAFNet demonstrates excellent performance on the binary classification task (Apnea/Normal) across Apnea-ECG Dataset and Huashan-Apnea Dataset. On Apnea-ECG dataset, it achieved a five-fold average accuracy of 0.9572, a sensitivity of 0.9575, a specificity of 0.9584, and an F1-score of 0.9557. On the Huashan-Apnea dataset, for the binary task, the model achieved an accuracy of 0.9003, a sensitivity of 0.8814, a specificity of 0.8314, and an F1-score of 0.8473.

Table 5 presents the per-class performance for the 4-class subtyping task. Despite the significant class imbalance, the proposed model demonstrates relatively balanced performance across different apnea subtypes.

**Table 5:** Per-class performance result in 4-class subtyping task.

Class	Acc	Precision	Sens	Spec	F1-score
OSA	0.8383	0.784	0.7568	0.8838	0.7702
CSA	0.9102	0.6706	0.7313	0.94	0.6996
MSA	0.872	0.7192	0.7306	0.9144	0.7249
HYP	0.8815	0.7835	0.7713	0.9219	0.7774

Specifically, OSA and HYP achieve relatively higher F1-scores (0.7702 and 0.7774, respectively), indicating robust detection performance for these more prevalent categories. For the minority classes, CSA and MSA also show competitive results, with F1-scores of 0.6996 and 0.7249, respectively. Although the performance on CSA is slightly lower, which can be attributed to its limited sample size, the model still maintains reasonable sensitivity (0.7313), suggesting its capability to identify minority pathological events.

**Figure 5:**
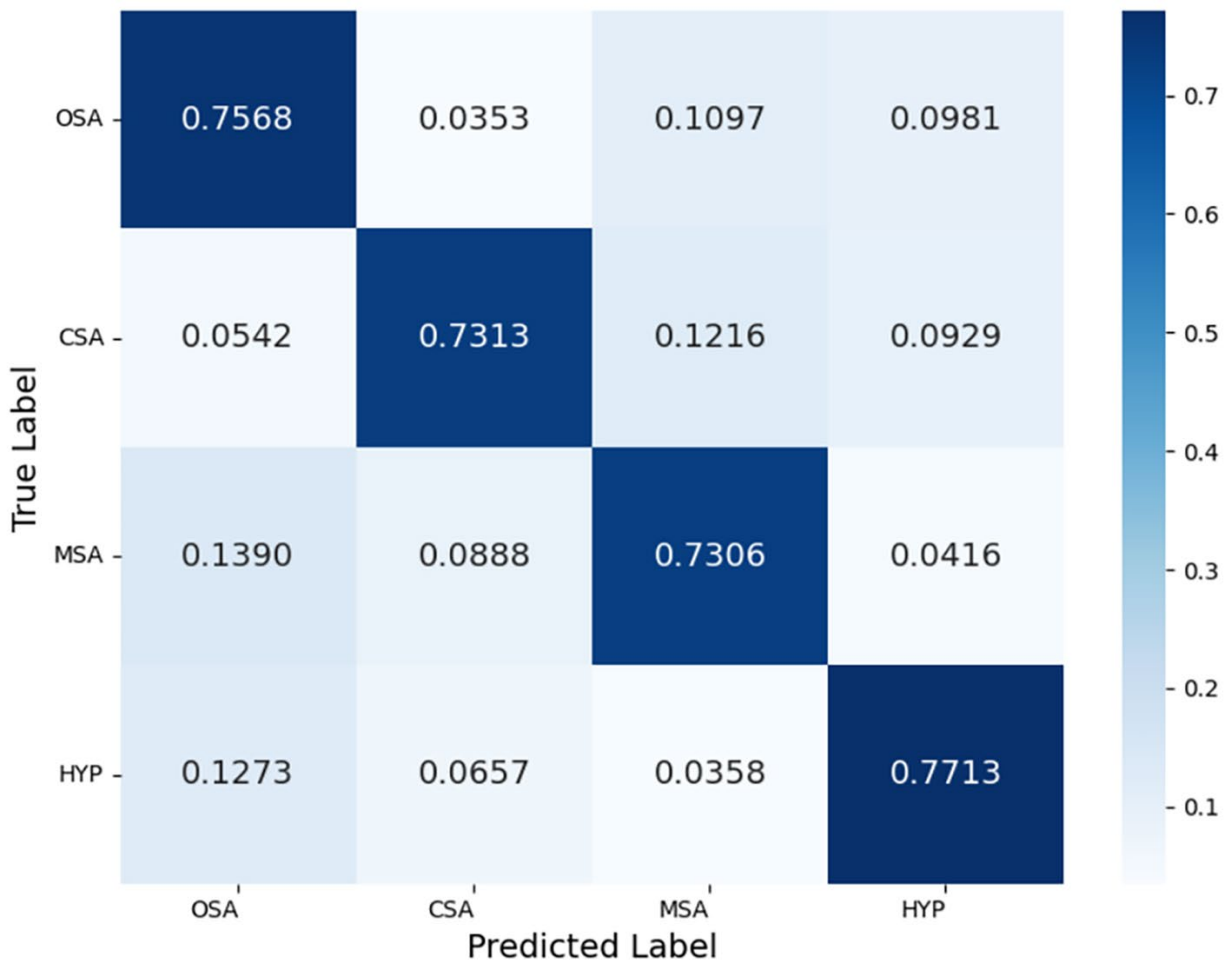
All fold confusion matrix in 4-class subtyping dataset.

Overall performance in sleep apnea subtyping: The results in Table 4 and Figure 5 show that DSCAFNet also achieves robust performance on the four-class classification task using Huashan-Apnea dataset, obtaining an accuracy of 0.7564, a sensitivity of 0.7546, a specificity of 0.9117, and an F1-score of 0.7497.

### 4.2 Comparative analysis of time-frequency representations: evaluating the impact of the synchrosqueezing transform

To systematically investigate the contribution of each component within our proposed feature engineering pipeline, we conducted a comprehensive ablation study. The performance of the network using different Time-Frequency Representations (TFRs) as input was evaluated on two distinct tasks: a binary apnea detection task (Normal vs. Apnea) across two datasets, and a more challenging four-class SA subtyping task on the Huashan-Apnea dataset. The detailed results are presented in Tables 6, 7, respectively.

**Table 6:** The performance of different TFRs as input in 2-class classification task in two datasets.

Different TFR input	Apnea-ECG				Huashan-Apnea			
	Acc	Sens	Spec	F1-score	Acc	Sens	Spec	F1-score
STFT	0.8732	0.8597	0.8953	0.8960	0.8453	0.8032	0.8253	0.8104
CWT	0.9106	0.8863	0.9135	0.9074	0.8676	0.8324	0.8343	0.8217
SST STFT	0.8955	0.8745	0.9087	0.9015	0.8635	0.8254	0.8632	0.8214
SST CWT	0.9325	0.9034	0.9245	0.9129	0.8867	0.8523	0.8445	0.8356
SST STFT & SST CWT	0.9572	0.9575	0.9584	0.9557	0.9003	0.8814	0.8314	0.8473

**Table 7:** The Performance of different TFRs input in 4-class subtyping task in Huashan-Apnea dataset.

Different TFR input	Acc	Sens	Spec	F1-score
STFT	0.6924	0.6855	0.8325	0.6858
CWT	0.7075	0.7064	0.8554	0.7147
SST STFT	0.7025	0.6924	0.8528	0.7139
SST CWT	0.7338	0.7297	0.8905	0.7354
SST STFT& SST CWT	0.7564	0.7546	0.9117	0.7497

Analysis of these results reveals several consistent and compelling trends. First, in both binary and four-class settings, the Continuous Wavelet Transform (CWT) consistently surpasses the Short-Time Fourier Transform (STFT) as a baseline representation. For instance, in the four-class task (Table 6), CWT achieves an accuracy of 0.7075, compared to 0.6924 for STFT. This underscores the superiority of CWT’s multi-resolution analysis in capturing the diverse and non-stationary patterns present in ECG signals across different apnea subtypes.

Second, the application of the Synchrosqueezing Transform (SST) as a post-processing step consistently enhances the performance of both baseline TFRs. This effect is particularly pronounced for CWT. As shown in Table 6, applying SST to CWT (SST CWT) boosts the accuracy from 0.7075 to 0.7338, a substantial improvement that highlights the value of sharpening TFRs to improve feature discriminability, especially when the classification task is more complex.

Most critically, the fusion of the two SST-enhanced representations (SST STFT & SST CWT) yields a significant performance leap, decisively outperforming all single-representation models across all evaluated tasks and datasets. In the binary classification on Apnea-ECG (Table 6), this dual-stream approach achieves a state-of-the-art accuracy of 0.9572. This superior performance is even more evident in the demanding four-class subtyping task (Table 7), where the fused input achieves an accuracy of 0.7564, representing a marked improvement of over 2.2% compared to the best single TFR (SST CWT).

These findings provide robust validation for our methodological choices. They demonstrate not only that SST-enhanced representations are richer in diagnostic information but also that SST-CWT and SST-STFT contain complementary features. The substantial performance gain from their fusion confirms that our dual-stream architecture is essential for capturing the comprehensive set of biomarkers required for both accurate apnea detection and fine-grained subtype classification.

### 4.3 Comparative analysis of different fusion strategies

A critical innovation of our DSCAFNet architecture is the Cross-Attention Fusion Module (CAFM), designed to synergistically integrate the dual time-frequency streams. To isolate and validate the contribution of this module, we conducted an ablation study, benchmarking its performance against two common fusion strategies: simple feature concatenation (Concat) (Liang et al., 2019) and a channel-wise gating mechanism (Chen et al., 2022). The comparative results for both binary and four-class classification tasks are detailed in Table 8.

**Table 8:** The results of different fusion method.

Method	2-Class Classification				4-Class Subtyping			
	Acc	Sens	Spec	F1-score	Acc	Sens	Spec	F1-score
Concat (2019)	0.8975	0.8785	0.8321	0.8454	0.7437	0.7329	0.8993	0.7313
Channel-wise gating (2022)	0.8814	0.8522	0.8157	0.8328	0.7369	0.7251	0.8876	0.7254
CAFM (Proposed Method)	0.9003	0.8814	0.8314	0.8473	0.7564	0.7546	0.9117	0.7497

The results unequivocally demonstrate the superiority of the proposed CAFM. In the binary classification task, CAFM achieves the highest performance across all metrics, reaching an accuracy of 0.9003 and an F1-score of 0.8473. This represents a clear improvement over the concatenation method, which is the next-best competitor.

Crucially, the performance gap widens considerably in the more challenging four-class subtyping task. Here, CAFM achieves an accuracy of 0.7564 and an F1-score of 0.7497, surpassing the concatenation method by a substantial margin of 1.27% in accuracy and 1.84% in F1-score. This highlights that simplistic fusion strategies, which are agnostic to the inter-dependencies between feature streams, are insufficient for distinguishing between the subtle patterns of different SA subtypes. In contrast, the cross-attention mechanism within CAFM allows the model to dynamically learn the correlations between the SST-CWT and SST-STFT representations, enabling one stream to selectively amplify the most salient information from the other.

This experiment results confirm that the architectural design of CAFM is a key factor in the overall success of the DSCAFNet model, particularly for fine-grained classification tasks that require a sophisticated understanding of complementary features.

## 5 Discussion

This study aimed to extract time-frequency dynamic features reflecting sleep apnea events from single-lead ECG signals and proposed a Dual Stream Cross Attention Fusion Network (DSCAFNet) based on synchrosqueezed time-frequency fusion.

To validate the effectiveness of the proposed DSCAFNet model, we compared our DSCAFNet model with several representative recent methods on both the public Apnea-ECG dataset and the private Huashan-Apnea dataset.

### 5.1 Performance comparison in public dataset

Table 9 presents a performance comparison of different algorithms, including traditional machine learning, 1D-CNNs, 2D-CNNs, and our method on the Apnea-ECG dataset. Overall, methods based on time-frequency maps (2D-CNNs) generally outperform models based solely on time-series data (1D-CNNs), indicating that joint time-frequency features can more effectively capture the non-stationary dynamics related to apnea in ECG signals.

**Table 9:** Comparison results in Apnea-ECG dataset.

Method category		Paper	Model	Acc	Sens	Spec	F1-score
Machine learning		Sinha and Das (2022)	Eigen spectrum	0.9435	0.9326	0.9579	0.94
		Fatimah et al. (2020)	Fourier composition	0.9259	0.8970	0.9467	—
		Feng et al. (2020)	Hidden markov	0.851	0.862	0.844	—
Deep learning	1D-CNN	Wang E. et al. (2023), Wang Y. et al. (2023)	1D-CNN-LSTM	0.8294	0.8125	0.8463	—
		Shen et al. (2021)	MSDA-1DCNN	0.894	0.898	0.891	—
		Chen et al. (2023)	BAFNet	0.9109	0.8869	0.9258	—
		Liu et al. (2023)	ApneaNet	0.9013	0.9514	0.8206	—
	2D-CNN	Niroshana et al. (2021)	Lightweight network	0.924	0.923	0.926	—
		Mashrur et al. (2021)	SCNN	0.9438	0.9430	0.9451	—
		Tang et al. (2025)	HRNet	0.942	0.926	0.959	0.942
		Proposed method	DSCAFNet	0.9572	0.9575	0.9584	0.9557

This enhancement is primarily attributed to the dual stream Cross-Attention Fusion Module, which enables the model to perform feature interaction between the two SST-enhanced spectral representations (CWT and STFT), thereby achieving complementary cross-spectral enhancement. Concurrently, the network’s hierarchical feature fusion strategy strengthens the energy concentration and temporal sensitivity at event boundaries, enabling the model to perform more robustly when distinguishing complex boundary samples, such as those between mild apnea and brief recovery periods.

In summary, our dual stream fusion strategy based on synchrosqueezed time-frequency features significantly enhances the detection accuracy and sensitivity for sleep apnea events, achieving state-of-the-art overall performance on this public dataset.

### 5.2 Performance comparison in Huashan-Apnea dataset

The experiments conducted on Huashan-Apnea dataset in Table 10 provide important evidence for the practical applicability and cross-domain generalization capability of the proposed DSCAFNet. For a fair comparison, the baseline models (e.g., VGG16, Simonyan and Zisserman (2014) and ResNet18, He et al. (2016)) were provided with the same SST-enhanced dual representations (SST-CWT and SST-STFT) as used in DSCAFNet. Specifically, the two time-frequency representations were combined via channel-wise concatenation to form a unified input, which was then fed into the baseline networks.

**Table 10:** Comparison of different classic 1D and 2D classification models in Huashan-Apnea dataset.

Models	Dimension	2-Class				4-Class			
		Acc	Sens	Spec	F1-score	Acc	Sens	Spec	F1-score
BAFNet (2023)	1D	0.8328	0.7546	0.8542	0.7519	0.6552	0.6268	0.6408	0.6139
VGG16 (2014)	2D	0.8542	0.7965	0.8402	0.8101	0.6824	0.6655	0.7894	0.6654
Resnet18 (2016)	2D	0.8793	0.8107	0.8867	0.8451	0.7186	0.7098	0.8369	0.7003
Proposed method	2D	0.9003	0.8814	0.8314	0.8473	0.7564	0.7546	0.9117	0.7497

In contrast, the proposed DSCAFNet employs a dual-stream architecture with a Cross-Attention Fusion Module (CAFM) to explicitly model the interactions between the two representations. Therefore, the performance improvements of DSCAFNet over the baseline models can be attributed to the more effective feature fusion strategy rather than differences in input representations.

Compared with the public Apnea-ECG dataset, Huashan-Apnea differs substantially in signal quality, recording hardware, and subject demographics, and further includes multiple apnea subtypes. The consistent performance advantages observed across these heterogeneous datasets indicate that the proposed model is able to extract stable and discriminative physiological patterns from single-lead ECG, even under varying acquisition conditions.

The model’s stable performance across these categories suggests that the dual stream architecture and feed-forward network (FFN) substantially enhance its capacity to model spectral–temporal complexity. In particular, the dual stream design allows the network to leverage complementary SST-enhanced CWT and STFT representations, while the cross-attention mechanism aligns these heterogeneous features to achieve semantic complementarity. This combination fosters richer high-level abstractions, enabling the model to better capture physiological signatures that differentiate, for example, central from mixed apnea.

Finally, the improved performance on multi-class clinical subtyping task emphasizes the model relevance for real-world diagnostic workflows. While most public datasets provide only binary annotations, the capability to differentiate among multiple apnea subtypes aligns more closely with clinical needs and has potential implications for personalized treatment planning. These findings collectively demonstrate that carefully designed time frequency transformations, coupled with cross-attention-based dual-stream fusion, can effectively capture autonomic and arrhythmic modulations reflected in ECG signals, thereby offering a promising technical route for automated and scalable sleep apnea screening.

### 5.3 Interpretability

**Figure 6:**
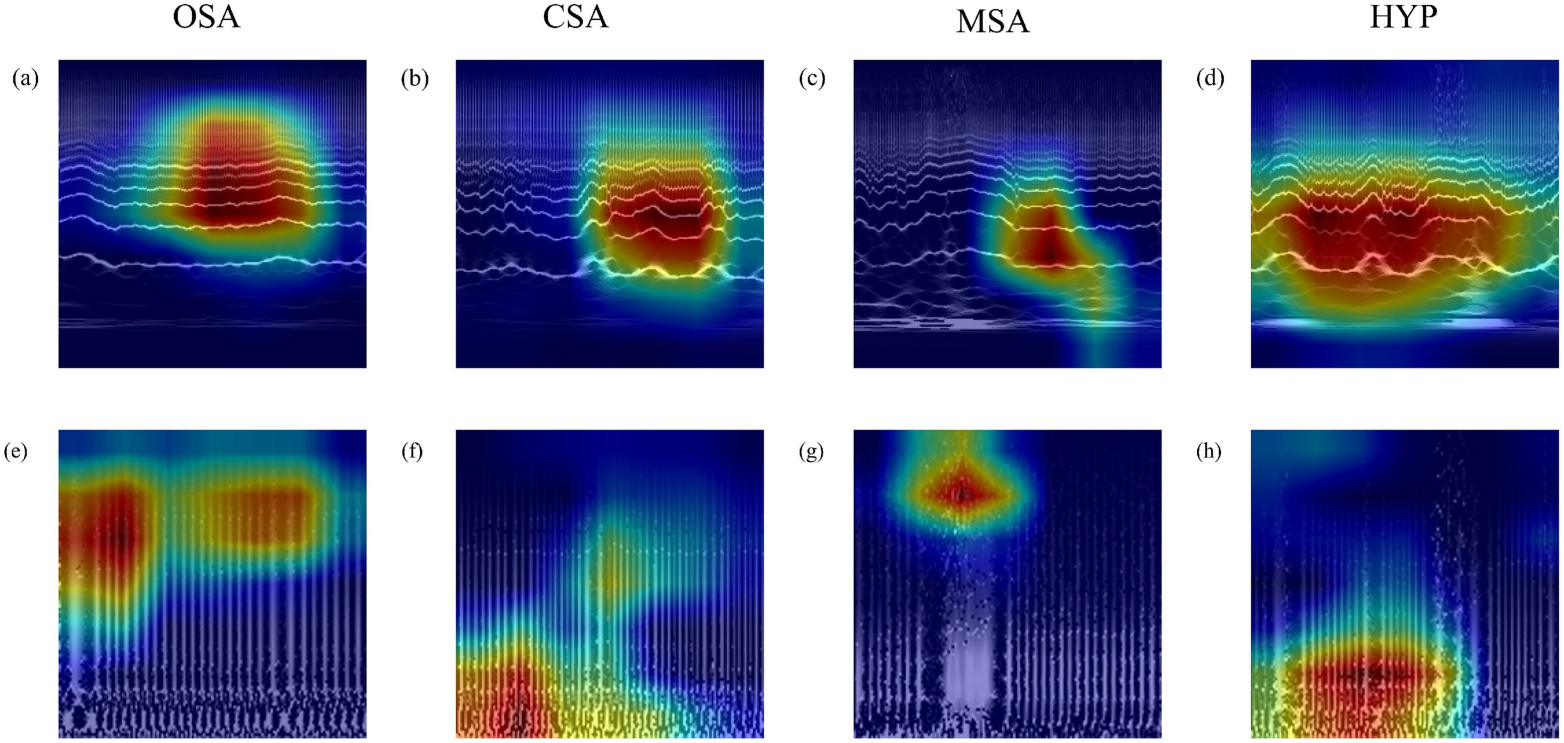
Grad-CAM visualizations of the proposed dual-stream network for the four sleep apnea categories (OSA, CSA, MSA, and HYP). The heatmaps illustrate the specific time-frequency regions prioritized by the model. **(a–d)** Class activation maps derived from the SCWT (Synchrosqueezed Continuous Wavelet Transform) stream. **(e–h)** The corresponding activation maps from the SSTFT (Synchrosqueezed Short-Time Fourier Transform) stream. Warmer colors (e.g., red and yellow) indicate regions with a higher contribution to the final classification decision, demonstrating the effectiveness of the cross-attention mechanism in capturing complementary multi-resolution features.

To further investigate the interpretability of the proposed framework, Grad-CAM was applied to visualize the discriminative regions in the time-frequency representations, as illustrated in Figure 6.

For the SST-CWT (SCWT) representations (Figures 6a–d), the model exhibits distinct attention patterns across different apnea subtypes. In particular, the attention for OSA samples tends to concentrate in relatively higher-frequency regions compared to CSA, while CSA shows more emphasis on lower-frequency components. Although some overlap exists between these patterns, the model is able to differentiate the dominant frequency characteristics associated with each subtype. For hypopnea events, the attention is more diffusely distributed and predominantly located in low-frequency regions, with a broader temporal span compared to other apnea types, suggesting a more prolonged and less abrupt physiological modulation.

For the SST-STFT representations (Figures 6e–h), clearer frequency-specific distinctions can be observed. OSA samples exhibit strong attention in higher-frequency bands, whereas CSA samples are primarily associated with lower-frequency regions. In contrast, MSA samples demonstrate attention across both low- and high-frequency components, reflecting the mixed nature of this subtype.

These observations highlight the complementary characteristics of the SST-CWT and SST-STFT representations. While SCWT captures more distributed and temporally extended patterns, SST-STFT provides sharper frequency localization. The integration of these two representations, together with the cross-attention fusion mechanism, enables the model to capture diverse and physiologically meaningful patterns associated with different sleep apnea events.

Overall, the Grad-CAM visualizations suggest that the proposed model does not rely on arbitrary features but instead focuses on discriminative time-frequency regions that are consistent with the underlying physiological differences among apnea subtypes, thereby supporting the interpretability of the framework.

### 5.4 Analysis of computing complexity

To further evaluate the computational cost of the proposed framework, we analyzed its model complexity and inference efficiency. The proposed DSCAFNet contains approximately 28 million parameters.

On an NVIDIA RTX 3060 GPU, the average inference time for a single epoch is approximately 13.3 ms. The computational overhead mainly arises from the dual SST-based time-frequency transformations and the dual-stream deep architecture with cross-attention.

While the current implementation may not be suitable for direct deployment on low-power edge devices, it can be effectively integrated into a cloud-based or offline diagnostic system, where data are collected using portable devices and processed on more powerful computing platforms.

### 5.5 Limitations

One limitation of this study is the significant gender imbalance in the Huashan-Apnea dataset (123 males vs. 10 females). Since sleep apnea may present differently across genders, this imbalance may introduce bias into the learned representations, potentially limiting the model’s generalizability to female populations.

As a result, the proposed model may be more sensitive to physiological patterns that are more prevalent in male subjects, while underrepresenting gender-specific characteristics in females.

Future work will focus on validating the model on more balanced datasets and exploring strategies to mitigate demographic bias, such as data augmentation and domain adaptation techniques.

## 6 Conclusion

This study introduces a Dual Stream Cross Attention Network (DSCAFNet) for sleep apnea (SA) detection from single-lead ECG. The method enhances time-frequency resolution using the Synchrosqueezing Transform (SST) and fuses complementary information from SST-enhanced Continuous Wavelet Transform (SST-CWT) and Short-Time Fourier Transform (SST-STFT) representations. Evaluated on the public Apnea-ECG dataset, DSCAFNet achieved an accuracy of 0.957 and an F1-score of 0.956 in binary classification. On private Huashan-apnea dataset, it attained accuracies of 0.900 for binary and 0.756 for four-class classification. The results demonstrate that the combination of SST enhancement and dual stream fusion effectively captures non-stationary dynamic features associated with sleep apnea, significantly improving both detection and classification performance. This work provides a feasible pathway for low-cost sleep apnea screening using wearable ECG technology.

## Data Availability

The datasets presented in this article are not readily available because the dataset contains is subject to privacy and ethical restrictions. Due to the informed consent signed by the participants and the regulations of the Institutional Review Board (IRB), the raw data cannot be made publicly available to protect patient confidentiality. De-identified data may be available from the corresponding author upon reasonable request. Requests to access the datasets should be directed to TZ, 23210720084@m.fudan.edu.cn.
